# Uremic Pruritus: From Diagnosis to Treatment

**DOI:** 10.3390/diagnostics12051108

**Published:** 2022-04-28

**Authors:** An-Yu Cheng, Lai-San Wong

**Affiliations:** Department of Dermatology, Kaohsiung Chang Gung Memorial Hospital and Chang Gung University College of Medicine, Kaohsiung 833, Taiwan; amandacheng26@gmail.com

**Keywords:** uremic pruritus, chronic kidney disease associated pruritus, chronic pruritus

## Abstract

Uremic pruritus, or chronic kidney disease-associated pruritus, is common, bothersome, and sometimes debilitating in patients with chronic kidney disease or end-stage renal disease. Due to its variable clinical manifestations, the diagnosis of uremic pruritus requires exquisite evaluation. Excluding itch resulting from other dermatological causes as well as other systemic conditions is essential for a proper diagnosis. The pathophysiology of uremic pruritus remains uncertain. Hypotheses including toxin deposition, immune system dysregulation, peripheral neuropathy, and opioid imbalance are supposed. This review summarizes the way to accurately diagnose uremic pruritus and describes the latest treatment options.

## 1. Introduction

Uremic pruritus, also known as chronic kidney disease-associated pruritus (CKD-aP), is a common, bothersome, and sometimes debilitating symptom in patients with chronic kidney disease (CKD) or end-stage renal disease (ESRD).

One large international cohort study of adult dialysis patients (Dialysis Outcomes and Practice Patterns Study (DOPPS)) reported that around 70% of hemodialysis patients suffered from pruritus and 40% of them were bothered by at least moderate pruritus. Notably, not only in patients with dialysis, the prevalence of moderate to severe pruritus in non-dialysis CKD patients was around 25% [1]. The prevalence of moderate to severe pruritus decreased from 46% in 1996 to 37% in 2015 [2,3], supposedly associated with the refinement of dialysis adequacy.

Risk factors of uremic pruritus in hemodialysis patients have been reported to be old age, gender, calcium–phosphate imbalance, longer duration of dialysis, and comorbidities such as concurrent cardiovascular disease, congestive cardiac failure, lung disease, liver disease, neurological disease, hepatitis C infection, and anemia [4]. However, some results were inconsistent in different studies [5,6,7,8]. Risk factors of developing pruritus in non-dialysis CKD patients include old age, female sex, advanced stage of CKD, lung disease, diabetes mellitus, and depression [1].

Pruritus in these patients dramatically affects their quality of life. Around half of them were bothered by pruritus all day long, and one-third were most bothered at night. The high percentage of night-time pruritus may lead to sleep disturbance. Sixty percent of patients with severe pruritus also frequently suffered from restless sleep [3]. Patients with pruritus also reported poor quality of life by appearance, mental distress, decreasing desire for social interaction, and ability to work. Notably, the bothersome symptoms not only decrease quality of life but also cause poorer medical outcomes. Pruritus in these patients was associated with significantly higher mortality, with a 13% higher risk in DOPPS phase I (1996–2001), a 21% higher risk in DOPPS phase II (2002–2004), and 37% higher risk in Japanese patients, and was suggested to be related to poor sleep quality [2,9]. One recent study using Taiwan’s national health insurance database revealed a higher risk of all-cause death and long-term morbidities including infection-associated hospitalization and major adverse cardiac and cerebrovascular events in patients with uremic pruritus [10,11].

Despite its high prevalence, disease burden, and even higher risk of mortality, pruritus is often underreported by patients and is underestimated by health care providers. Some 69% of medical facilities directors underestimated the prevalence of pruritus in their facilities, and 17% of patients who were frequently bothered by itchiness never reported their symptoms to any member of the medical facility. Among those who did report, they were more likely to report to nephrologists and staff of the dialysis facility, followed by dermatologists [3].

Therefore, raising awareness of uremic pruritus is crucial for both patients and health care providers. Health care providers should also be careful about making an accurate diagnosis in CKD patients with chronic pruritus. At the same time, understanding its pathophysiology to develop proper treatment is essential to improve patients’ quality of life and medical outcomes. In this review, we focus on current advances in diagnosis and treatment to overcome the unmet needs of patients with uremic pruritus.

## 2. Clinical Features

The clinical manifestations of uremic pruritus are variable. The severity varies from somewhat uncomfortable to extremely disturbing and inducing restlessness. About 20% of patients experienced being very much and extremely itchy in the DOPPS study [3]. The distribution is usually generalized and symmetric, but may be localized and is more common on the back, face, and shunt arm [12]. However, there is no dermatomal distribution. Pruritus is aggravated by dryness, heat, cold, stress, and showering. The skin is often lacking prominent lesions in the affected patients. However, in addition to xerosis, there may be skin lesions secondary to repetitive scratching, such as excoriation, crusts, impetigo, and prurigo nodularis. Most patients (61%) reported that pruritus has no relation to the timing of dialysis, but some reported that the pruritus was more severe during dialysis (15%), or soon after dialysis (9%), or on non-dialysis days (14%) [3].

Chronic pruritus also developed in patients receiving peritoneal dialysis, although it is less studied compared to patients with hemodialysis. A meta-analysis study showed comparable prevalence of CKD-aP in patients with hemodialysis and peritoneal dialysis (55% vs. 56%) [13]. However, Min et al. reported a higher prevalence of pruritus in patients with peritoneal dialysis compared to patients with hemodialysis (62.6% vs. 48.3%) [14]. The intensity of pruritus was higher in patients undergoing peritoneal dialysis and was associated with lower total weekly Kt/V, longer duration of dialysis, higher dietary protein intake, higher parathyroid hormone, and higher high-sensitivity C-reactive protein [13,15].

Several observations have shown that the prevalence of pruritus declined after renal transplantation [16,17]. A recent large study revealed that pruritus resolved completely in 73.7% (56/76) of renal transplant recipients [18]. Furthermore, pruritus improved or totally resolved in 97.3% of patients (74/76) with renal transplantation. Intriguingly, new onset of pruritus was found in 18.2% (22/121) of patients with renal transplantation with undetermined reasons [18].

## 3. Diagnosis

Uremic pruritus is defined by itching directly resulting from CKD, without other explainable conditions [19]. Due to its variability and the lack of specific skin lesions, there are no well-established criteria nor laboratory tests for the diagnosis of uremic pruritus. Accurate diagnosis of uremic pruritus may be challenging. Diagnosis requires thorough consideration including dermatologic, renal, hepatobiliary, endocrine, rheumatologic, hematologic or oncologic, neuropathic, and psychogenic causes. The initial approach is to evaluate whether there is primary or inflamed skin lesions to exclude other dermatological causes of pruritus. Common dermatological causes of chronic pruritus include atopic dermatitis, psoriasis, chronic urticaria, dermatophytosis, scabies infestations, and bullous pemphigoid [20]. To be noted, skin lesions secondary to scratching and rubbing, such as crusts, erosions, ulcerations, and prurigo nodularis, should not be counted. Personal drug history should also be reviewed to exclude drug-induced pruritus without visible skin lesions. Angiotensin-converting enzyme inhibitors, clonidine, calcium antagonists, beta blockers, diuretics, and allopurinol may induce itch by activating mu-opioid receptors [21]. Whether patients have chronic pruritus before deteriorating renal function helps to clarify the diagnosis of uremic pruritus. Referring to dermatologists may help to reach an appropriate diagnosis of uremic pruritus.

## 4. Pathogenesis

Generally, itch transmission can be defined as histaminergic and non-histaminergic pathways [22]. Due to the insufficient response to antihistamine treatment in uremic pruritus, the non-histaminergic pathway is suggested to be important in uremic pruritus. The pathogenesis of uremic pruritus is not completely understood. The possible pathogenesis of uremic pruritus is multifactorial and includes uremic toxins, immune dysregulation, neuropathy, and opioid imbalance [19,23,24,25,26,27].

The most studied potential pruritogens in the pathogenesis of uremic pruritus include histamine, calcium, phosphate, magnesium, parathyroid hormone, and vitamin A [28,29,30,31]. Uremic toxins, substances accumulating due to impaired renal function, are supposed to participate in uremic pruritus. This is supported by evidence of decreased severity and prevalence of uremic pruritus with the improvement in dialysis efficiency [18,19]. Uremic toxins are further classified as water-soluble small molecules, protein-bound compounds, and middle molecules [32]. Among them, beta-2 microglobulin, indoxyl sulfate, and p-Cresyl sulfate draw researchers’ attention to their possible roles in uremic pruritus [33]. However, a recent metabolomic analysis failed to show different concentrations of water-soluble small molecules in hemodialysis patients with and without itch [34].

The immune system may also participate in the pathogenesis of uremic pruritus. Studies comparing hemodialysis patients with and without uremic pruritus showed significantly enhanced T helper 1 cells and serum interleukin (IL)-6, IL-2, and IL-31 levels in patients with uremic pruritus [23,24,25,35]. Partial responses with immunomodulatory treatments such as calcineurin inhibitors, phototherapy, and mast cell stabilizers in uremic pruritus indicate the role of subclinical inflammation in uremic pruritus [26,36].

Both peripheral neuropathy and central neuropathy are believed to participate in uremic pruritus. One study showed a reduction in innervation in the skin of patients with uremic pruritus [37]. The association of uremic pruritus and restless leg syndrome in dialysis patients and successful treatment with gabapentin implies the role of neuropathy in uremic pruritus [19]. In addition, increasing evidence suggests that both central and peripheral opioid receptors contribute to uremic pruritus. Among three types of opioid receptors, mu-opioid receptor agonists induce itching, and kappa-opioid receptor agonists attenuate itching. Delta-opioid receptor has a limited impact on itching [27]. Recent successful trials of nalbuphine, nalfurafine, and difelikefalin support the hypothesis of opioid system imbalance in uremic pruritus [38,39,40].

## 5. Treatment

Due to the limited understanding of the pathogenesis of uremic pruritus, current treatments for uremic pruritus remain elusive. There was no Food and Drug Administration (FDA)-approved treatment for uremic pruritus until difelikefalin was approved in the United States in 2021 [41,42]. Conventional treatments including emollient, topical agents, antihistamine, dialysis modification, phototherapy, and serotonin receptor antagonists are reviewed here. Recently, more evidence suggests that gabapentin, pregabalin, opioid receptor agonists and antagonists, and biologics play roles in uremic pruritus. We summarize updated treatment options for uremic pruritus in this review. (Table 1)

### 5.1. Moisturizers

Xerosis is found in 50–85% of patients with uremic pruritus and is an aggravating factor of pruritus [43]. Multiple trials using different emollients including glycerol and paraffin, 10% urea and dexpanthenol, physiological lipids, and baby oil decrease xerosis and pruritus in patients with uremic pruritus [44,45,46,47,48]. Emollient is suggested as first-line therapy in patients with uremic pruritus, especially for those with less severity [19].

### 5.2. Topical Calcineurin Inhibitor (Tacrolimus, Pimecrolimus)

Tacrolimus, a calcineurin inhibitor, is used for its anti-inflammatory effects in atopic dermatitis and vitiligo [49,50]. A case study reported that 0.03% tacrolimus ointment reduced pruritus intensity in three cases with severe uremic pruritus [51]. However, a randomized, double-blind, vehicle-controlled study demonstrated that 0.1% tacrolimus applied twice daily for 4 weeks was not more efficacious than the vehicle [52]. Another 8-week, randomized, double-blind study of 1% pimecrolimus also revealed a lack of efficacy compared to the placebo group [53].

### 5.3. Other Topical Agents

Capsaicin, a compound found in chili pepper and transient receptor potential vanilloid member 1 (TRPV1) agonist, was used to relieve pain and neuropathy [54]. Three randomized controlled trials of capsaicin cream for uremic pruritus showed limited efficacy [55]. Pramoxine is a topical anesthetic with antipruritic effects. A randomized, double-blind, controlled comparative trial with 28 patients using 1% pramoxine lotion showed a 61% decrease in pruritus intensity compared to 12% in the placebo group [56].

### 5.4. High Quality of Dialysis

Since uremic toxins are suggested as potential pruritogens, increasing the efficiency of dialysis and modification of hemodialysis prescription is a potential strategy for the treatment of uremic pruritus. One randomized trial found that increased dialysis efficiency with mean Kt/V up to 1.28 resulted in pruritus improvement compared to mean Kt/V of around 1.09 [57]. Dialysis modification to remove more middle molecules also showed improvement in pruritus intensity. Hemodialysis modifications including high-flux hemodialysis [58], hemodiafiltration with hemodialysis [59], and high-permeability hemodialysis [60] have shown significant decreases in pruritus intensity. Therefore, increasing dialysis efficiency as well as modulating dialysis parameters are suggested as first-line treatments in patients with uremic pruritus [12].

### 5.5. Phototherapy

Phototherapy has been widely used in inflammatory skin diseases such as psoriasis, atopic dermatitis, and vitiligo [49,50,61] by modulating Th1 and Th2 lymphocyte differentiation and attenuating Th1-mediated responses [62,63]. Broadband ultraviolet B phototherapy was found to be effective in patients with uremic pruritus compared to ultraviolet A phototherapy [64]. One single-blind, randomized, controlled trial of 21 patients with uremic pruritus showed a significant improvement in pruritus by narrowband ultraviolet B phototherapy and long-wave ultraviolet A phototherapy compared to the non-treated group [62]. A recent controlled trial of narrowband ultraviolet B phototherapy revealed a significant reduction in visual analog scale (VAS) from 9.1 to 1.9 compared to treatment with antihistamine and emollients. In addition, the effect was sustained for up to 6 months in most of the study group [65]. Due to its wide adoption in pruritic dermatoses, narrowband ultraviolet B is considered an efficacious treatment in uremic pruritus.

### 5.6. Antihistamine

Antihistamine, mainly targeting histamine H1 receptor, has been widely used for anti-pruritus. It has been reported that over half of physicians prescribed an oral anti-histamine and one-fourth of them prescribed a topical anti-histamine as first-line therapy for uremic pruritus; however, anti-histamines were generally unsatisfactory for the treatment of uremic pruritus [3,19,66]. In addition, side effects of anti-histamines such as dizziness, sedation, and urine retention are concerns in patients with CKD [19].

### 5.7. Gabapentin, Pregabalin

Both pregabalin and gabapentin, analogs of gamma-aminobutyric acid, are modulators of neurotransmitters, acting possibly by decreasing neurotransmitter release [67]. The neuropathic role was implicated in the pathogenesis of various pruritic disorders such as brachioradial pruritus and pruritus in patients with diabetic neuropathic pain [68]. Several clinical trials of gabapentin and pregabalin have shown them to be statistically significant in the reduction in pruritus intensity in patients with uremic pruritus [69,70,71,72]. One recent systematic review showed decreased severity of pruritus after treatment with gabapentin in four out of seven studies (*n* = 171). Incidences of adverse effects with gabapentin including dizziness, drowsiness, and somnolence were higher but not significant in a pooled analysis (*n* = 290) [73]. Another clinical trial demonstrated that pruritus was relieved in 85% of 71 patients by gabapentin or pregabalin and that patients intolerant to gabapentin might tolerate pregabalin [74]. One study comparing the effects of gabapentin after each dialysis session and pregabalin daily showed a significant improvement in the reduction in pruritus and neuropathic pain in both groups [72]. A systematic review concluded that gabapentin is the most reliable and effective treatment as an off-label treatment for uremic pruritus [75].

### 5.8. Opioid Receptor Agonist/Antagonist

#### 5.8.1. Naloxone

Intravenous injection of naloxone, a mu-receptor antagonist, was firstly reported to be efficacious in treating uremic pruritus in 1984 [76]. However, inconsistent results were found in subsequent large studies of oral naltrexone for uremic pruritus. In addition, some studies revealed frequent adverse effects such as nausea and sleep disturbance [77,78,79].

#### 5.8.2. Nalfurafine

Nalfurafine, a selective kappa agonist, was found to be effective in uremic pruritus in a multicenter, randomized, double-blind, placebo-controlled clinical study [80]. A phase III randomized, double-blind, placebo-controlled study of 337 patients also revealed that nalfurafine significantly reduce the itch severity in intractable uremic pruritus [38]. Nalfurafine was officially approved for clinical use for uremic pruritus in Japan.

#### 5.8.3. Difelikefalin

Recently, difelikefalin, a peripheral restricted and selective kappa opioid receptor agonist, proved its efficacy in the treatment of uremic pruritus in a large double-blind, placebo-controlled, multicenter phase III trial [40]. A total of 378 hemodialysis patients with moderate to severe pruritus were randomly treated with intravenous difelikefalin at the dose of 0.5 μg per kilogram or placebo for 12 weeks. Difelikefalin significantly decreased the intensity of pruritus (at least 3 points from baseline according to a 24-h worst itching intensity numerical rating scale) in 51.9% of patients compared to 30.9% of patients in the placebo group. Itch-related quality of life also improved significantly compared to the placebo group. Common adverse events include diarrhea, dizziness, and vomiting, but no adverse events of dysphoria, hallucination, euphoria, or discontinuation-related discomfort were reported in the difelikefalin group. Based on the successful results of the phase III trial, difelikefalin was approved by the FDA in the United States in 2021. A regulatory review is ongoing in Europe and a phase III trial is in progress in Japan. Oral difelikefalin is under investigation [41].

#### 5.8.4. Nalbuphine

Nalbuphine, a combination of kappa-opioid receptor antagonist and mu-opioid receptor agonist, has been reported to be beneficial for morphine-related itch [81]. In addition, nalbuphine also decreased the intensity of uremic pruritus [39]. A multicenter, randomized, double-blind, placebo-controlled trial of 373 hemodialysis patients with moderate to severe pruritus demonstrated that the group receiving nalbuphine 120 mg twice daily for 8 weeks reported significantly decreased pruritus. However, there was no significant difference between nalbuphine at the dose of 60 mg twice daily and the placebo group.

### 5.9. Mast Cell Stabilizer

Mast cell stabilizers, which prevent degranulation of inflammatory mediators from mast cells, have been shown to be effective in uremic pruritus, and include topical cromolyn sodium [82], oral cromolyn sodium [83], ketotifen [70], and zinc sulfate [84]. One double-blind, randomized clinical trial of 52 patients revealed similar efficacy in decreasing pruritus severity and no significant difference in adverse effect with gabapentin and ketotifen [70]. However, one randomized control trial of 36 patients with 4-week duration of zinc sulfate showed non-significant reduction in pruritus in hemodialysis patients [85]. Mast cell stabilizer is safe and potentially efficacious in uremic pruritus, but more studies are needed.

### 5.10. Montelukast 

Leukotriene B4, primarily released by macrophage and leukocytes, is involved in itch and may induce scratching [86,87]. Montelukast, a leukotriene receptor antagonist, is used in atopic dermatitis, asthma, allergic rhinitis, and idiopathic urticaria. One randomized double-blind controlled trial of 80 hemodialysis patients receiving 10 mg montelukast daily for 30 days revealed that montelukast significantly decreased pruritus compared to the placebo group. The authors concluded that montelukast might serve as an add-on treatment in intractable uremic pruritus [88].

### 5.11. Serotonin Receptor Antagonist: Ondansetron

5-HT3 receptor antagonists have been studied for their treatment efficacy in uremic pruritus. Ondansetron, a selective 5-HT3 receptor antagonist, had an insignificant effect on uremic pruritus in two randomized controlled trials [89,90].

### 5.12. Nemolizumab

Due to the higher concentration of IL-31 in hemodialysis patients with uremic pruritus, the role of nemolizumab, an IL-31 receptor alpha antibody, in the treatment of uremic pruritus is suggested [23]. A randomized, double-blind, placebo-controlled phase IIB trial of a single dose of nemolizumab was conducted in 69 patients with uremic pruritus but failed to meet the primary efficacy endpoint [91].

### 5.13. Dupilumab

As the possible involvement of IL-31 was implicated in uremic pruritus, the role of T-helper 2, which is the upstream regulator of IL-31, in uremic pruritus has been suggested. Dupilumab, an IL-4 receptor alpha-blocker, was reported to successfully treat cases with intractable uremic pruritus [92,93]. More studies are necessary.

### 5.14. Acupuncture, Acupressure

Acupuncture, defined by acupuncture needle insertion into specific points of the skin as treatment, has long been used for a variety of symptoms, such as acute or chronic pain, sleep disturbance, and poor quality of life in East Asia [94]. Acupuncture was believed to act through modulating the endogenous opioid system [95], which accounts for the hypothesis of it treating uremic treatment. Similarly, acupressure stimulates the acupuncture points with body parts of practitioners or designed equipment. A systematic review including six trials showed that acupuncture and acupressure were effective for uremic pruritus [96]. Recent studies comparing Zolpidem and acupressure revealed that both improved sleep quality and quality of life in patients with uremic pruritus-associated sleep disturbance [97,98]. However, more evidence is needed to affirm this as a recommended treatment for uremic pruritus. 

### 5.15. Charcoal 

Given the hypothesis of non-dialyzable uremic toxins as possible pruritogens, the adequate removal of potential toxins by charcoal is a reasonable therapeutic option. Activated charcoal, a non-selective absorbent, is usually used for detoxication in certain kinds of poisoning. An 11-patient, placebo-controlled, double-blind, crossover study showed that a daily dose of 6 g of activated charcoal for 8 weeks decreased pruritus in most patients with uremic pruritus [99]. In addition, coated charcoal in extracorporeal techniques showed decreased levels of parathyroid hormone and pruritus in a study of 12 patients [100]. Although evidence with large case numbers and well-designed studies are lacking, a recent review showed a promising role of charcoal in uremic pruritus [101].

### 5.16. Other Treatment

Early studies have proposed the association of uremic pruritus with hyperthyroidism, calcium, and phosphate. It is reported that the level of calcium multiplied by phosphate is correlated with the extent of pruritus after parathyroidectomy, and intractable pruritus improved after parathyroidectomy in some cases [102,103].

Thalidomide, an immunomodulator and neuromodulator, was initially observed to reduce pruritus in dialysis patients with leprosy [104]. One early randomized, crossover study with 29 patients found a statistically significant decrease in pruritus in the thalidomide group [105].

Gamma-linolenic acid (GLA), an essential acid, is thought to modulate T lymphocytes and lymphokines. In one small-sized, randomized control trial, GLA-enriched cream significantly improved the pruritus severity in dialysis patients [106].

Cannabinoids act on the endocannabinoid system to modulate pruritus and shed light on the treatment of chronic and refractory pruritus with legalized medical marijuana [107]. In a study of 21 patients applying topical cream containing structured physiological lipids, anandamide, and palmitoylethanoamine twice daily for 3 weeks, 8 out of 21 patients with uremic pruritus were completely free from pruritus [45]. However, randomized trials for cannabinoids are lacking [108].

Kidney transplantation, a kidney replacement therapy, largely decreased or cured chronic pruritus in patients with uremic pruritus [12,18,109]. Other medical considerations remain issues due to the shortage of human kidneys.

Catabolism of exogenous protein may lead to the retention of protein-bound molecules. Therefore, protein-restrictive diets and probiotics may serve as a potential treatments for uremic pruritus [101]. Omega-3 supplements were found to be effective to decrease pruritus in a cross-randomized trial [110].

**Table 1:** Summary of studies of interventions for uremic pruritus.

Authors	Study Design	Participants	Enrollment	Intervention	Comparator	Efficacy
Duque et al. (2005) [52]	Randomized, double-blind, vehicle-controlled study	HD	N = 22	0.1% tacrolimus ointment twice daily for 4 weeks	Vehicle	No significant effect.
Ghorbani et al. (2011) [53]	Randomized double-blind study	Not mentioned	N = 60	Pimecrolimus 1% twice daily for 8 weeks	Placebo	No significant effect.
Ko et al. (2011) [62]	Single-blind, randomized, controlled trial	HD, PD, CKD	N = 21	NB-UVB phototherapy three times a week for 6 weeks	Long-wave UVA radiation	Significant and comparable improvement in the VAS scores in both groups.
Sherjeena et al. (2017) [65]	Controlled trial	CKD, stage IV, V	N = 30	NBUVB phototherapy every 3 days for 15 sessions	Topical liquid paraffin, 10 mg oral cetirizine daily	Significant effect. VAS 9.13 to 1.9 at 3 months (NBUVB). VAS 9.1 to 8.8 at 3 months (control).
Mapar et al. (2015) [85]	Pilot randomized, triple-blind study	HD	N = 36	Zinc sulfate 220 mg daily for 4 weeks	Placebo	No significant effect.
Mahmudpour et al. (2017) [88]	Randomized double-blind controlled trial	HD	N = 80	Montelukast 10 mg daily for 30 days	Placebo	Reduction in VAS score was significantly greater in the montelukast group (2.73) compared to placebo group (5.47).
Amirkhanlou (2016) [70]	Double-blind randomized clinical trial	HD	N = 52	Gabapentin 100 mg daily for 2 weeks	Ketotifen 1 mg twice daily for 2 weeks	Significant reduction in both groups (88.4% in gabapentin group vs. 76.9% in group ketotifen group).
Eusebio-Alpapara et al. (2020) [73]	Meta-analysis	HD	N = 315	Gabapentin 100 mg daily, 100–400 mg 2–4 times per week	Antihistamine, pregabalin, placebo	Gabapentin decreased the pruritus severity compared to the placebo (*n* = 171).
Kumagai et al. (2010) [38]	Randomized, double-blind, placebo-controlled study	HD	N = 337	Nalfurafine hydrochloride 5 μg, 2.5 μg for 14 days	Placebo	Significant reduction in VAS in both dosages of nalfurafine compared to placebo.
Mathur et al. (2017) [39]	Randomized, double-blind, placebo-controlled trial	HD	N = 373	Nalbuphine 120 mg, 60 mg twice daily for 8 weeks	Placebo	Significant effect with nalbuphine 120 mg, but not with nalbuphine 60 mg.
Fishbane et al. (2020) [40]	Double-blind, placebo-controlled, phase III trial	HD	N = 378	Intravenous difelikefalin 0.5 μg per kilogram per week for 12 weeks	Placebo	A decrease of at least 3 points (WI-NRS score) in 49.1% of the difelikefalin group (27.9% of the placebo group).
Kinugasa et al. (2021) [91]	Randomized, double-blind, placebo-controlled clinical study	HD	N = 69	Nemolizumab 0.125, 0.5, 2.0 mg/kg on day 1	Placebo	No significant effect.

HD, hemodialysis; PD, peritoneal dialysis; CKD, chronic kidney disease; VAS, visual analog scale; NB, narrow band; WI-NRS, worst itch numeric rating scale. The data were retrieved by 25 March 2022.

## 6. Proposed Algorithm from Diagnosis to Treatment

**Figure 1:**
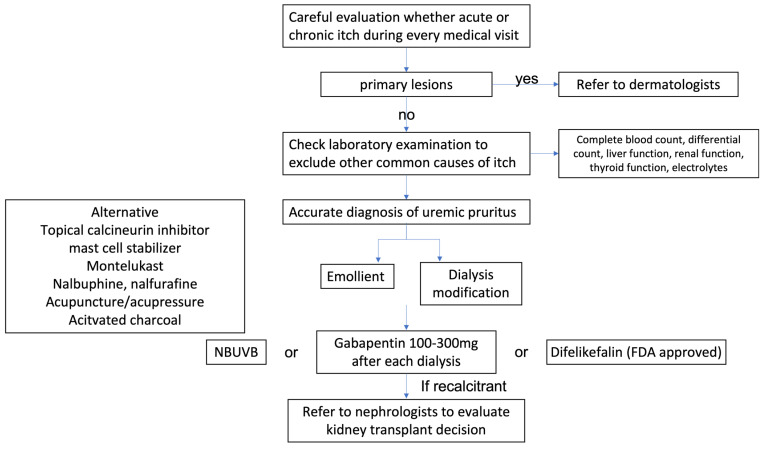
Proposed diagnosis and treatment algorithm of uremic pruritus.

**Figure 2:**
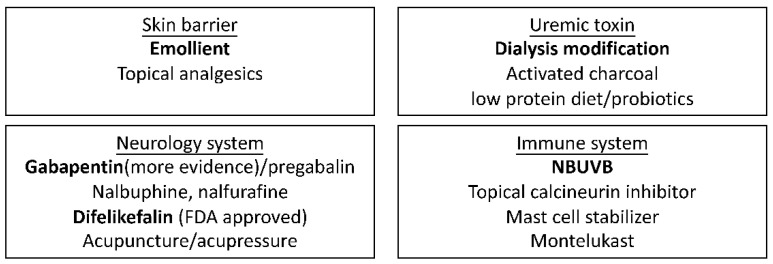
Treatment options for uremic pruritus classified by possible targeted etiologies. The bold ones have greater evidence in current literature.

Based on the updated evidence, we propose the diagnosis and treatment algorithm in CKD patients (Figure 1). Firstly, any pruritus and skin condition should be evaluated in patients with CKD every visit. If primary skin lesions or acute pruritus are present, physicians may refer patients to dermatologists to exclude other common causes of itch. Screening laboratory examinations including complete blood count, differential count, liver function test, renal function test, thyroid function, and electrolytes should also be arranged to exclude other systemic etiologies. Once an accurate diagnosis of uremic pruritus is made, physicians may initiate an emollient for skin rehydration. The dialysis efficacy should also be re-evaluated to increase efficiency and correct electrolytes in case of imbalance. Modification with dialysis membrane is suggested to remove middle molecules and protein-bound molecules [111]. Difelikefalin was the first FDA-approved medicine for uremic pruritus in the United States. Gabapentin, NBUVB, and difelikefalin may be considered as first-line treatments. Mast cell stabilizers, montelukast, nalbuphine, nalfurafine, acupuncture or acupressure, and activated charcoal may be considered as alternative therapies. Diet adjustment with low protein and probiotics also serve as potential treatment strategies. Treatment options are listed and classified according to potential targeted etiologies (Figure 2). Kidney transplantation is considered in extreme cases.

## 7. Conclusions

In this review, we summarized the current evidence on diagnosis and treatment of uremic pruritus. Given the limitations of proper diagnosis of uremic pruritus, we proposed an algorithm for more accurate diagnosis of uremic pruritus. In summary, a complete assessment to exclude occult itch origin and early diagnosis of uremic pruritus are essential. Emollients and dialysis modification are general preventive guidance in the treatment of uremic pruritus. Gabapentin, phototherapy, and FDA-approved difelikefalin are promising treatment options for recalcitrant uremic pruritus. Other developing treatments are opioid mediators, neural transmission mediators, and biologics targeting IL-31. Due to the high prevalence and unsatisfactory treatment response, more research on uremic pruritus should be prioritized.

## Data Availability

Not applicable.
